# The efficacy and safety of elbasvir/grazoprevir treatment in HCV genotype 1 patients in Taiwan

**DOI:** 10.1002/jmv.25605

**Published:** 2019-10-22

**Authors:** Tzu‐Cheng Tsai, Shin‐Tarng Deng, Chao‐Wei Hsu

**Affiliations:** ^1^ Department of Pharmacy Chang Gung Memorial Hospital Taoyuan Taiwan; ^2^ Department of Long Term Care Hsin Sheng College of Medical Care and Management Taoyuan Taiwan; ^3^ Department of Gastroenterology and Hepatology Chang Gung Memorial Hospital Taoyuan Taiwan; ^4^ Chang Gung University College of Medicine Taoyuan Taiwan

**Keywords:** elbasvir/grazoprevir, HCV genotype 1, hepatitis C virus, sustained the virologic response

## Abstract

**Background:**

Elbasvir/grazoprevir (EBR/GZR) is a new generation, fixed‐dose, combination antiviral drug used in chronic hepatitis C virus (HCV) genotype (GT) 1 or 4 infection. Our study evaluates the clinical efficacy and safety of EBR/GZR after its launch in Taiwan.

**Methods:**

This is a retrospective observational study. Patients who had received EBR/GZR for chronic HCV GT 1 between June 2017 and April 2018 were recruited. Patients’ age, sex, HCV GT, changes in HCV RNA level before and after treatment, sustained virologic response 12 weeks (SVR12) after the cessation of drug administration, side effects, and interaction effects were used to evaluate the clinical efficacy and safety.

**Results:**

A total of 149 patients were recruited. Of them, 145 (97.3%) had HCV GT 1b, and the rest had HCV GT 1a; most of the EBR/GZR‐related side effects in this study were mild. Three participants were discontinued because their alanine transaminase levels were elevated to over 10 times the upper limit of normal. The therapeutic effect analyses revealed a rapid virologic response rate of 95.3% and an SVR12 rate of 98%. Subgroup analyses performed using SVR12 as the outcome variable revealed three demographic factors HCV GT 1, hepatocellular carcinoma medical history, and noncirrhosis plus HCV RNA level.

**Conclusions:**

This study confirmed that EBR/GZR is safe and effective for treating patients with HCV GT 1 and exhibited excellent overall clinical efficacy in Taiwan. The therapeutic effects are unrelated to factors such as sex, HCV RNA level before treatment, and history of liver cirrhosis.

## Highlight

- EBR/GZR is safe and effective for HCV GT 1 in Taiwan.
- SVR rate is high and compare with western country.
- Medication related side effect is mild and tolerable.
- There are no significant statistic difference in renal function in each group during the period of treatment and follow‐up.

## 1 INTRODUCTION

The infection of liver cells by hepatitis C virus (HCV) can result in the inflammation and necrosis of these cells, which can further result in the development of liver cirrhosis or fibrosis and ultimately lead to hepatocellular carcinoma (HCC). Currently, six HCV genotypes (GTs) are known, with most infections being GT 1 (49.1% of the global HCV‐infected population), followed by GT 3 (17.9%), GT 4 (16.8%), GT 2 (11.0%), and GT 5 or GT 6 (<5%).^1^ The prevalence of hepatitis C is approximately 4.4% in Taiwan.^2^ Populations with HCV GT 1b are mainly found in North America (26%), Latin America (39%), Europe (50%), and Asia. In Japan, approximately 65% of patients with HCV have GT 1b. In Taiwan, it is estimated that 600 000 people have HCV. Among them, approximately 53% have HCV GT 1 infection, 40% have HCV GT 2 infection, and the remaining proportion have HCV GT 3 to GT 6 infections.^3^

Since 2011, various direct‐acting antiviral (DAA) drugs have been successively launched in the the United States, Europe, and Japan markets. DAA inhibits the NS3/4A protease, the NS5A protein, or the RNA polymerase, which are crucial in the life cycle of HCV. Two of the most notable DAAs are boceprevir and telaprevir. The sustained virologic response (SVR) can be as high as 90%. Nevertheless, the DAA is considerably expensive. Furthermore, they must be used with interferon and ribavirin (RBV), and they are poorly tolerated and often elicit severe side effects.^3^, ^4^, ^5^ However, since interferon‐free DAAs were developed and launched in 2014, they have superseded other treatments for chronic hepatitis C patients. Nowadays, we have several DAAs to treat HCV infection, such as sofosbuvir base, glecaprevir/pibrentasvir, elbasvir/grazoprevir (EBR/GZR), and others. Some DAAs have limitations due to renal insufficiency or protease inhibitor‐related hyperbilirubinemia. Thus, we decided to study ERB/GZB because of its simple use and did not concern for renal function issue; in addition, patient compliance might be adequate.^6^, ^7^

EBR/GZR is a fixed‐dose combination DAA composed of 50 mg of EBR and 100 mg of GZR. EBR is an NS5A inhibitor and GZR is an NS3/4A protease inhibitor and was approved by the US Food and Drug Administration in 2016 for chronic HCV GT 1 or GT 4 infection regardless of whether they have received pegylated interferon alfa (PEG‐IFN) plus RBV treatment previously.^1^, ^5^, ^8^, ^9^, ^10^, ^11^ The results of phase 3 random assignment clinical trials such as C‐EDGE TN, C‐EDGE TX, and C‐SURFER have revealed that EBR/GZR has excellent therapeutic effects in chronic hepatitis C patients. The SVR12 (HCV RNA is undetectable at week 12) rates for these three groups were 95%, 92%, and 94%, respectively.^1^, ^9^, ^10^, ^11^ Subsequently, it has been listed as a novel DAA covered by National Health Insurance (NHI) in Taiwan since August 2017. This policy has benefited numerous patients with hepatitis C and has encouraged patients to actively seek treatment.

The aim of this study is to evaluate the clinical efficacy and safety of EBR/GZR based on clinical data collected after the introduction of EBR/GZR to the study site hospital in 2017 in Taiwan. In addition, it is verified if these results in Taiwan match those reported in clinical trials such as C‐EDGE TN and C‐EDGE TX.

## 2 MATERIALS AND METHODS

### 2.1 Design

This study is a retrospective observational study and was approved (IRB number: 201800718B0) by the Institutional Review Board of Chang Gung Medical Foundation. The data used were sourced from the Chang Gung Research Database (CGRD) of the Lin‐Kou Chang Gung Medical Center.

#### 2.1.1 The International Classification of Diseases: 10th revision, clinical modification

International Classification of Diseases: 10th revision, clinical modification (ICD‐10‐CM) diagnostic codes and laboratory examination data from the database were used to identify patients who had received outpatient EBR/GZR treatment for chronic hepatitis C between 1 June 2017 and 30 April 2018. Subsequently, the clinical efficacy and safety during the treatment period were evaluated based on the following factors: HCV GT, HCV RNA, liver and renal function, blood biochemical values, side effects, and drug interaction effects.

### 2.2 Sample

*Inclusion criteria*: Outpatients with chronic HCV GT 1 who fit the inclusion criteria were identified using ICD‐10‐CM codes, laboratory examination codes, and NHI data. The applicable diagnostic codes were B18.2 (chronic viral hepatitis C) and Z22.52 (carrier of viral hepatitis C). The applicable laboratory examination codes were L72‐955 (anti‐HCV Ab), M23‐108 (HCV GT [Roche ver 2.0]), M23‐088 (HCV GT), and M23‐079 (HCV RNA). Liver cirrhosis defined by Fibroscan Fibrosis‐4 (FIB‐4) or ultrasound. If Fibroscan was more than 12 kPa or FIB‐4 (age [years] × AST [U/L])/(PLT [109/L] × ALT [U/L]1/2) was great than 6.5 or liver cirrhosis by ultrasound with splenomegaly or endoscope showed esophageal/gastric varices defined as cirrhosis by our guidance of DAA reimbursement by NHI in Taiwan. For those using EBR/GZR tablets at their own expense or those who fulfilled the criteria of the NHI Executive Plan for Coverage of All‐Oral Hepatitis C Drugs (with liver fibrosis level F3 or F4), the EBR/GZR NHI treatment combination was used to treat HCV GT 1a infection using EBR/GZR without or with RBV for 12 weeks (without drug‐resistant virus strains) or 16 weeks (with drug‐resistant virus strains) and to treat HCV GT 1b infection using EBR/GZR with or without RBV for 12 weeks (the applicable order codes are HCVDAA0005, HCVDAA0006, and HCVDAA0007, respectively).

*Exclusion criteria*: Patients with chronic HCV GT 2, GT 3, and GT 4; patients with chronic HCV with a coinfection of HIV (applicable ICD‐10‐CM diagnostic code: B20 [HIV/HCV coinfection]).

### 2.3 Data collection

The medical research database of the Lin‐Kou Chang Gung Medical Center was used to perform data clustering and analysis. Approval was granted to use the following data sets for research purposes basic demographic information files, outpatient diagnostic files, outpatient expense quotation files, laboratory examination result files, medical and medication order files, and drug‐related files.

*Monitored items*: Patient age and sex, measured anti‐HCV, HCV RNA level, aspartate aminotransferase or alanine aminotransferase (ALT) level, bilirubin level, blood urea nitrogen, creatinine, albumin, HCV GT, resistance‐associated substitution, and complete blood count, which includes laboratory examination results related to white blood cell counts, hemoglobin level, and platelet count, before EBR/GZR administration, 4, 12, or 16 weeks after drug administration and 12 weeks after the treatment course ended.

*Evaluations of clinical efficacy and safety*: SVR12 analysis, measured 12 weeks after the cessation of drug intake; analysis of inappropriate prescriptions; analysis of drug interaction effects; types and severity of side effects. In addition, data were collected on drugs that could cause interaction effects when used concomitantly with EBR/GZR, such as carbamazepine, phenytoin, rifampicin, rifater, and cyclosporine. Finally, this study also collected data on the side effects and the corresponding treatment measures. The severity and the corresponding treatment measures were mild (no medication is given), moderate (medication administered), and severe (cessation of EBR/GZR treatment or hospitalization).

### 2.4 Analysis methods

Analyses in this study were mainly conducted using descriptive statistics. If the dependent or outcome variable was continuous, the data were represented as mean ± standard error and analyzed using Student *t* test; if the dependent or outcome variable was categorical, the data were represented as a numerical value and percentage and analyzed using the χ^2^ method. Significance was set at *P* < .05.

## 3 RESULTS

**Figure 1:**
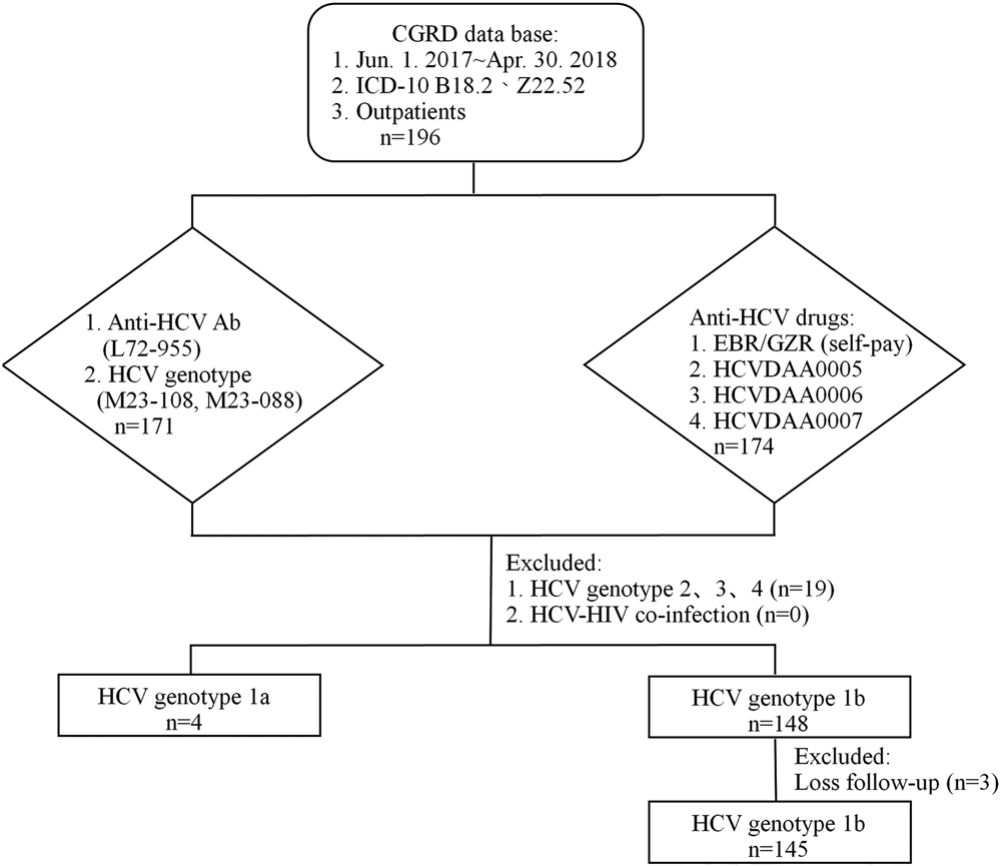
Study flowchart

The participant recruitment process is illustrated in the flowchart in Figure 1. Using the data from the CGRD, 196 patients with ICD‐10‐CM diagnostic codes B18.2 and Z22.52 were identified from the data between 1 June 2017 and 30 April 2018. Among these 196 patients, hepatitis C antibodies and HCV GTs were detected in 171 patients. Medications were prescribed for 174 patients. Nineteen patients with HCV GT 2, GT 3, and GT 4 were excluded, and three with HCV GT 1 were lost to follow‐up. A total of 149 outpatients who fit the inclusion criteria remained. Of the149 participants recruited, 82 were female (55.0%). Regarding age distribution, 99 participants were aged 65 years or older (66.4%); the average age was 69.0 ± 10.7 years. Most participants have GT 1b infection (n = 145; 97.3%); only four have GT 1a infection (no drug‐resistant strains detected: n = 3; unexamined: n = 1). In terms of medical history, 79 participants (53%) reported a history of liver cirrhosis, whereas 27 participants reported switching to EBR/GZR treatment after failure to respond to PEG‐IFN plus RBV treatment previously. Finally, 88 participants (59.1%) had an HCV RNA viral load higher than 800 000 IU/mL. The average HCV RNA viral load among participants was 2 523 970 IU/mL (Table 1).

**Table 1:** Baseline characteristics and comparisons with liver cirrhosis or noncirrhosis in HCV patients

Parameter	Overall (n = 149)	Cirrhosis (n = 79)	Noncirrhosis (n = 70)	*P* value
Age (mean ± SD)	69.0 ± 10.7	71.6 ± 9.3	66.1 ± 11.4	.0014
<65, n(%)	50 (33.6)	20 (25.3)	30 (42.9)	.0236
≧65, n(%)	99 (66.4)	59 (74.7)	40 (57.1)	
Gender, n (%)				.0708
Male	67 (45.0)	41 (51.9)	26 (37.1)	
Female	82 (55.0)	38 (48.1)	44 (62.9)	
HCV genotype 1, n (%)				.0563
1a	4 (2.7)	4 (5.1)	0 (0)	
1b	145 (97.3)	75 (94.9)	70 (100)	
HCV treatment history, n (%)				.5751
Naive	122 (81.9)	66 (83.5)	56 (80.0)	
Prior IFN‐based treatment	27 (18.1)	13 (16.5)	14 (20.0)	
HCV RNA (mean), IU/mL	2 523 970	2 198 326	2 891 483	.2502
≧800 000 IU/mL, n (%)	88 (59.1)	41 (51.9)	47 (67.1)	.0589
<800 000 IU/mL, n (%)	61 (40.9)	38 (48.1)	23 (32.9)	
History of HCC, n (%)				.0011
Yes	27 (18.1)	22 (27.8)	5 (7.1)	
No	122 (81.9)	57 (72.2)	65 (92.9)	
HBV coinfection, n (%)	9 (6.0)	6 (7.6)	3 (4.3)	.3974
HIV coinfection, n (%)	0 (0)	0 (0)	0 (0)	NA
Child‐Pugh score, n (%)				
Class A: 5	64 (43.0)	64 (81.0)	…	NA
Class A: 6	15 (10.0)	15 (19.0)	…	NA
Class B	0 (0)	0 (0)	…	NA
Class C	0 (0)	0 (0)	…	NA
ALT (mean ± SD), IU/mL	86.1 ± 94.1	94.4 ± 76.5	70.9 ± 71.0	.0542
Total bilirubin (mean ± SD), mg/dL	0.8 ± 0.5	0.9 ± 0.4	0.8 ± 0.6	.3073
Albumin (mean ± SD), g/dL	4.04 ± 0.37	3.89 ± 0.35	4.22 ± 0.31	<.0001
CKD (eGFR), mL/min/1.73 m^2^	89.6 ± 38.0	86.4 ± 37.2	93.3 ± 38.5	.2689
Stage 1, n (%)	74 (49.7)	36 (45.6)	38 (54.3)	.2882
Stage 2, n (%)	46 (30.9)	25 (31.6)	21 (30.0)	.8282
Stage 3, n (%)	17 (11.4)	10 (12.7)	7 (10.0)	.6105
Stage 4, n (%)	2 (1.3)	2 (2.5)	0 (0)	.1802
Stage 5, n (%)	10 (6.7)	6 (7.6)	4 (5.7)	.6470
Comorbidity, n (%)				
Diabetic mellitus	12 (8.1)	8 (10.1)	4 (5.7)	.3233
Coronary artery disease	4 (2.7)	2 (2.5)	2 (2.9)	.9024
Hypertension	32 (21.5)	18 (22.8)	14 (20.0)	.6795
CKD stage 4 & 5	12 (8.1)	8 (10.1)	4 (5.7)	.3233
Gastritis, ulcer, GERD	19 (12.8)	5 (6.3)	14 (20.0)	.0125
Hyperlipidemia	7 (4.7)	3 (3.8)	4 (5.7)	.5810
Respiratory disease	4 (2.7)	2 (2.5)	2 (2.9)	.9024
Malignancy	8 (5.4)	4 (5.1)	4 (5.7)	.8603
Benign prostatic hyperplasia	7 (4.7)	5 (6.3)	2 (2.9)	.3175
Hypothyroidism	3 (2.0)	1 (1.3)	2 (2.9)	.4901
Others	12 (8.1)	8 (10.1)	4 (5.7)	.3233

Abbreviations: ALT, alanine aminotransferase; ESRD, end stage renal disease; GERD, gastroesophageal reflux disease; HCC, hepatocellular carcinoma; HCV, hepatitis C virus; IFN, interferon.

**Figure 2:**
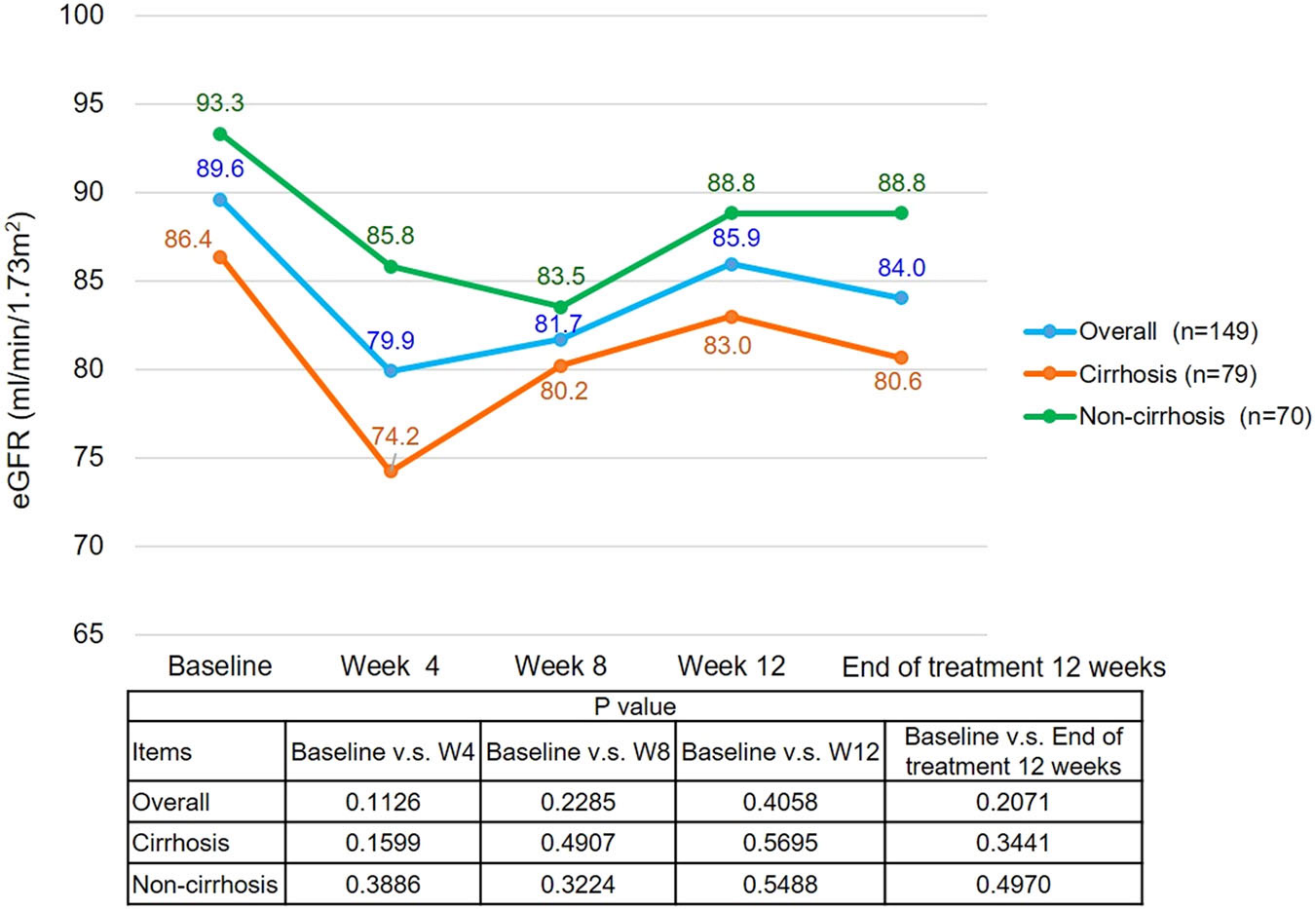
Time‐course changes in the eGFR level

Regarding the analysis of coinfections related to HCV, 9 participants (6.0%) had a coinfection of hepatitis B, none had a coinfection of HIV, and 27 (18.1%) had comorbidity with HCC. During EBR/GZR treatment, 76 patients (51%) required concurrent medication treatments for other chronic diseases. Among them, 18 patients had two comorbidities and 13 had three comorbidities. The comorbidities were mainly chronic diseases, including hypertension (n = 32); gastrointestinal diseases such as gastritis, gastroesophageal reflux disease, and gastric ulcer (n = 19); diabetes mellitus (n = 12); chronic kidney disease and kidney failure (n = 12); and hyperlipidemia (n = 7) (for details, refer to Table 1 and Figure 2).

Between‐group comparisons using liver cirrhosis medical history as the between‐group factor revealed the following: (a) the average age of those in the cirrhosis group (71.6 ± 9.3 years) was significantly higher than that of those in the noncirrhosis group (66.1 ± 11.4 years; *P* = .0014), and (b) the proportion of patients with a history of HCC was significantly higher in the cirrhosis group than in the noncirrhosis group (27.8% vs 7.1%; *P* = .0011). No significant differences were observed for the between‐group differences of the cirrhosis and noncirrhosis group on other outcome variables such as sex, HCV GT, HCV RNA viral load, and hepatitis C treatment history (Table 1).

In terms of the interaction effects between concomitant medications and EBR/GZR, most prescriptions were reasonable, and the medications showed no obvious interaction effects with each other. Only four cases of established drug interaction effects of EBR/GZR with statin drugs (n = 3 for atorvastatin; n = 1 for simvastatin) were noted. The average dosage for atorvastatin was <10 mg/day and the average length of concomitant use was 51.3 days; the average dosage for simvastatin was <10 mg/day and the average length of concomitant use was 77 days.

Side effects and associated treatment measures: side effects were reported in 45 patients (30.2%). Elevated bilirubin level (7.4%) was the most common side effect, followed by fatigue (4.0%), insomnia (4.0%), dizziness (4.0%), elevated ALT level (3.4%), headache (2.7%), and itchy skin (2.0%). Regarding associated treatment measures, no medication was administered in 32 of the affected patients (21.5%) and medication was prescribed in nine patients (6.0%); administration of EBR/GZR was stopped in three patients (2.0%), and no participant was hospitalized because of side effects. The proportion of patients who experienced the side effect of elevated bilirubin level was significantly higher in the cirrhosis group than in the noncirrhosis group (12.7% vs 1.4%; *P* = .0089) (Table 2).

**Table 2:** EBR/GZR‐related adverse events

Adverse events (AE) (n)(%)	Overall (n = 149)	Cirrhosis (n = 79)	Noncirrhosis (n = 70)	*P* value
≧1 AEs	44 (30.2)	26 (32.9)	18 (25.7)	.3365
Total bilirubin elevation	11 (7.4)	10 (12.7)	1 (1.4)	.0089
Insomnia	6 (4.0)	3 (3.8)	3 (4.3)	.8797
Dizziness	6 (4.0)	4 (5.1)	2 (2.9)	.4942
Fatigue	6 (4.0)	2 (2.5)	4 (5.7)	.3240
ALT elevation	5 (3.4)	3 (3.8)	2 (2.9)	.7504
Headache	4 (2.7)	1 (1.3)	3 (4.3)	.2550
Pruritus	3 (2.0)	2 (2.5)	1 (1.4)	.6323
Skin reaction	1 (0.7)	1 (1.3)	0 (0)	.3449
Nausea	1 (0.7)	0 (0)	1 (1.4)	.2865
Dry mouth	1 (0.7)	0 (0)	1 (1.4)	.2865
Management of AE, n (%)				
Observation	32 (21.5)	18 (22.8)	14 (20.0)	.6795
Medication	9 (6.0)	6 (7.6)	3 (4.3)	.3974
Drug discontinuation	3 (2.0)	2 (2.5)	1 (1.4)	.6323

*Note*: Total bilirubin normal range ≦ 1.3 mg/dL; ALT normal range ≦ 36 U/L.

Abbreviations: ALT, alanine aminotransferase; EBR/GZR, elbasvir/grazoprevir.

A medication‐related increase in ALT levels was reported in five patients. The involved patients had an average age of 66.2 years and all had GT 1b infection; three had a history of liver cirrhosis and only one had ever received PEG‐IFN plus RBV treatment. On average, the side effect of elevated ALT levels occurred at 8.2 weeks after the participant started taking EBR/GZR. Of the five reported cases, four patients experienced elevated ALT levels 10 times greater than the normal range: three of them stopped taking EBR/GZR because of this, and the other patient continued with the medication due to the lack of obvious symptoms. The time taken for elevated ALT to return to normal levels varies from person to person. In this study, an average of 90.6 days (range, 22‐246 days) was required for the elevated ALT levels of the participants to return to normal. Nonetheless, HCV RNA was not detected in these five patients who experienced elevated ALT levels when measurements were taken at week 12 after the cessation of EBR/GZR treatment. EBR/GZR‐related elevation of bilirubin level was reported in 11 patients, 10 of which had a history of liver cirrhosis, and 4 (36.4%) out of these, 11 participants experienced this side effect within 2 weeks of commencing EBR/GZR. On average, the side effect of elevated bilirubin levels occurred 4.9 weeks after the EBR/GZR. All patients exhibited only mild levels of bilirubin elevation with no symptoms noted. Therefore, EBR/GZR was continued until the treatment course was completed. For four of the involved patients, their bilirubin levels still did not return to the normal range, even 3 months after the completion of the treatment cycle. Notably, all of them had liver cirrhosis.

According to the regulations of the NHI Executive Plan for Coverage of All‐Oral Hepatitis C Drugs, for patients receiving medication, their HCV RNA viral load levels should be measured 4 weeks after drug administration, directly after the treatment course has ended, and 12 weeks after the treatment course has ended. This is to monitor the changes in HCV RNA viral load to evaluate the therapeutic effect of the drug. In this study, one participant experienced the side effect of itchy skin within 1 week of the administration of EBR/GZR, and the participant completed the rest of the treatment using ledipasvir/sofosbuvir instead of EBR/GZR. Therefore, this patient was excluded from the therapeutic effect analysis, resulting in a final analysis sample of 148 patients. The analysis results revealed that 4 weeks after the administration of EBR/GZR, HCV RNA can no longer be detected in the blood serum of 141 patients, thus demonstrating a rapid virologic response (RVR) rate of 95.3%. Measurements taken directly after the treatment course ended revealed that HCV RNA could no longer be detected in the blood serum of 146 patients, indicating an end of treatment virologic response (EOTVR) rate as high as 98.6%. At 12 weeks after the cessation of treatment,145 patients exhibited an SVR, thus achieving an SVR12 rate of 98.0%. (Three patients who still did not have their HCV RNA levels examined at the time were excluded. Among them, the condition of one patient had deteriorated, and the doctors in charge did not order an HCV RNA laboratory examination for the other two patients).

**Figure 3:**
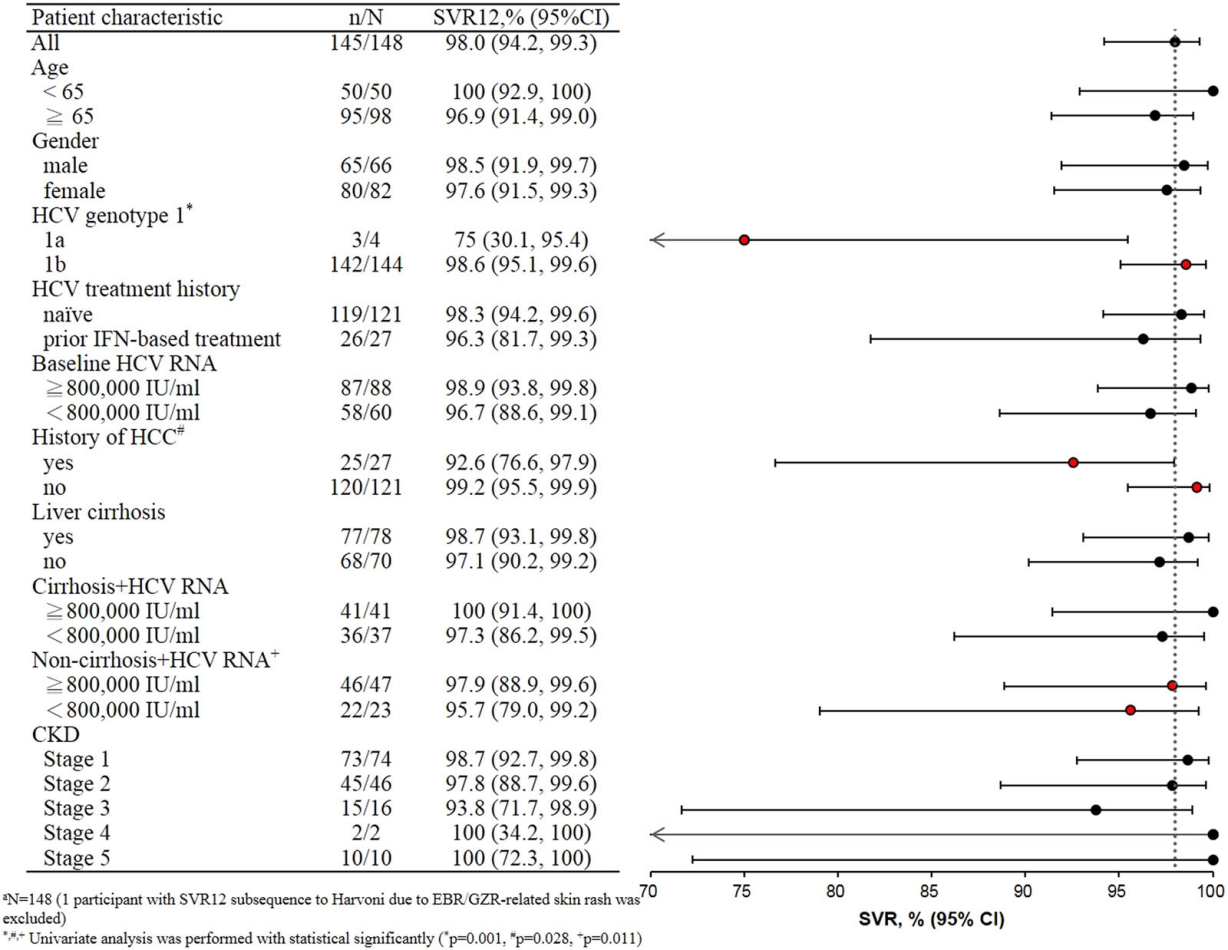
Sustained virologic response 12 weeks subgroup analysis

Subsequently, subgroup analyses were performed using SVR12 as the outcome variable. The results of the univariate analyses revealed that three demographic factors exhibited significant differences in terms of SVR12 rates, namely HCV GT 1 subtype (GT 1a vs GT 1b; *P* = .001), medical history of HCC (HCC vs non‐HCC; *P* = .028), and noncirrhosis plus HCV RNA viral load (≧800 000 vs <800 000 IU/mL; *P* = .011). No significant between‐group differences in terms of SVR12 rate were noted for any of the other demographic factors analyzed (ie, sex, age, cirrhosis history, and HCV RNA viral load level before treatment; Figure 3).

## 4 DISCUSSION

The prevalence of hepatitis C is approximately 4.4% in Taiwan. It is estimated that 600 000 people have HCV. Among them, approximately 53% have HCV GT 1 infection, 40% have HCV GT 2 infection, and the remaining proportion have HCV GT 3 to GT 6.^3^ In this study, analysis results revealed an RVR rate of 95.3%, an EOTVR rate as high as 98.6% and an SVR rate of 98.0%. Between‐group comparisons using a history of liver cirrhosis revealed that those in the cirrhosis group were significantly older than those in the noncirrhosis group and the percentage of patients with a medical history of HCC was significantly higher in the cirrhosis group than in the noncirrhosis group. No significant between‐group differences between the cirrhosis and noncirrhosis groups were observed for sex, HCV RNA viral load level, and hepatitis C treatment history.

EBR/GZR is metabolized by the liver, both are transformed into substrates for the cytochrome P450 3A (CYP3A) protein. Therefore, using EBR/GZR concomitantly with strong inducers and inhibitors of the CYP3A protein is prohibited. This is to prevent the concentration of EBR/GZR from being lowered or raised, which subsequently leads to the attenuation or enhancement of therapeutic effects.^1^, ^11^ According to related studies, when EBR/GZR is used concomitantly with drugs such as carbamazepine, phenytoin, rifampicin, rifater, and cyclosporine, prominent interaction effects occur.^12^ Most prescriptions for the majority of patients in this study were reasonable. The only exception was the four patients who used EBR/GZR concomitantly with statin drugs. According to relevant studies, when statin drugs are used concomitantly with EBR/GZR, the dosage of atorvastatin should not exceed 20 mg/day, whereas the dosage for rosuvastatin should not exceed 10 mg/day. As for fluvastatin, lovastatin, and simvastatin, the lowest recommended dosage should be administered. This is to prevent EBR/GZR from enhancing the concentration of statin drugs. In addition to administering these drugs, the patients’ conditions should be closely monitored to timely detect any statin‐related side effects.^12^ In the current study, four patients concomitantly used statin drugs and EBR/GZR. Although the average length of concomitant use was more than 50 days, the average daily dosage was ≦10 mg/day. No significant drug interaction effects were observed, and the relevant lipid profile values were within a reasonable range.

According to the results of large‐scale clinical trial studies, such as C‐EDGE TN, C‐EDGE, TEC‐CORAL, and CO‐INFECTION,^2^, ^8^, ^10^, ^11^ the usage of EBR/GZR elicits mostly mild or moderate side effects. The most common side effects include headache (17%), fatigue (15%), and nausea (9%). The most crucial side effects of EBR/GZR are the elevation of ALT (1%) and bilirubin (2.2%) levels. It is similar to other protease inhibitors, EBR/GZR usage carries the risk of abnormal liver functioning. Most reported cases of ALT elevation were mild (grade 1). Severe ALT elevation (ie, an ALT level at least five times the upper limit of the normal range [5 × ULN]), were only observed in less than 1% of the patients taking EBR/GZR. On average, ALT elevations occur after 8 weeks of medication and asymptomatically. Most ALT levels gradually returned to the normal range as they proceed with treatment or after the treatment course. However, if symptoms are noted after ALT elevation or when ALT elevation is accompanied by elevated bilirubin levels, alkaline phosphatase, or international normalized ratio, EBR/GZR should be stopped. In addition, it should be stopped when a patient's ALT level is more than 10 × ULN according to the instruction of medicine or physician concern the safety issue. In our study, only three patients' discontinuous EBR/GZR may be related to protease inhibitors. Therefore, patients’ liver function should be monitored both before and during the treatment course.^4^, ^10^, ^11^ Related studies have posited that the risk of ALT elevation is related to the concentration of grazoprevir; the higher the concentration of grazoprevir is, the higher the elevation risk is. ALT elevation is relatively unrelated to the presence of cirrhosis and the length of EBR/GZR treatment.^11^ Elevation of bilirubin caused by EBR/GZR usually occurs 2 weeks after the drug is taken and usually decreases gradually along the treatment course.^11^ Bilirubin elevation is not necessarily related to the presence of liver cirrhosis and changes in liver function.^2^

In this study, side effects were reported in 45 patients. Among the reports, elevated bilirubin level was the most common, followed by fatigue, insomnia, dizziness, and increased ALT level. In most patients, the side effects were mild; only nine patients (6.0%) needed medication to relieve these side effects. EBR/GZR‐related elevation of the bilirubin level was reported in 11 patients. On average, elevated bilirubin levels occurred 4.9 weeks after the EBR/GZR. All patients exhibited only mild bilirubin levels elevation and continued until the treatment course was completed. For four of the involved patients, their bilirubin levels still did not return to the normal range—this may be related to pre‐existing liver fibrosis status.

According to the results of the C‐EDGE TN phase 3 random assignment clinical trial, during a 12‐week course of EBR/GZR in naive patients with HCV GT 1, GT 4, and GT 6, the overall SVR12 rate was as high as 95%. The SVR12 rate for the cirrhosis subgroup was 97.1% and that for the noncirrhosis subgroup was 93.9%. The SVR rate for patients with an HCV RNA viral load of ≦800 000 IU/mL before treatment was as high as 100%, while an SVR rate of 92.3% was observed for patients who had an HCV RNA viral load of >800 000 IU/mL before treatment.^9^, ^10^ The C‐EDGE TX clinical trial targeted patients with HCV GT 1, GT 4, and GT 6 who had received PEG‐IFN plus RBV treatment but failed to respond to it. These participants received EBR/GZR with or without RBV treatment for 12 weeks, and the results revealed that the overall SVR12 rate of this treatment was slightly higher than for EBR/GZR only (94% vs 92%). However, the probability of the occurrence of side effects was higher for the combined treatment group, with a 10% to 20% chance that patients would experience the side effect of anemia (hemoglobin <10 g/dL). In our study, the results of the univariate analyses revealed that three demographic factors exhibited significant differences in terms of SVR12 rates, namely HCV GT 1 subtype, medical history of HCC, and noncirrhosis plus HCV RNA viral load and no significant between‐group differences in terms of SVR12 rate were noted for any of the other demographic factors analyzed (Figure 3). The cases number of GT 1a and medical history of HCC were limited and might result in bias; otherwise, the noncirrhosis plus HCV RNA viral load might be due to the difference of cases number (23 patients <800 000 IU/mL and 47 patients ≧800 000 IU/mL).

EBR/GZR is mainly excreted through feces (>90%); the proportion of it excreted through the kidneys is usually less than 1%. Therefore, the dosage need not be adjusted when patients with impaired kidney functioning (eGFR <30 mL/min/1.73 m^2^). The results of a C‐SURFER clinical trial in HCV patients with comorbid stage 4 or 5 kidney impairment confirmed its therapeutic effects (SVR12, 94%) and is highly safe.^8^ In 2018, researchers from the United States and Italy each published research evaluating and discussing the clinical efficacy of EBR/GZR after it was launched in these nations.^13^, ^14^ TRIO is a retrospective study conducted collaboratively by physicians across 34 US states. The study sample consisted of 470 patients with HCV GT 1 and GT 4. The overall SVR12 rate for patients with HCV GT 1 was 99%. Subgroup analyses on GT 1 revealed the following results: the SVR12 rate for patients with stage 4 or 5 kidney impairment was 99%, and the SVR12 rate of patients in the noncirrhosis group was higher than that for the cirrhosis group (100% vs 96%).^13^ The aforementioned Italian study on the efficacy and safety of EBR/GZR had a considerably smaller sample size than this study (29 patients with HCV). In terms of clinical efficacy, an SVR12 rate of 100% was reported. The clinical safety evaluation revealed that common side effects such as fatigue or headache were not observed. However, four participants experienced asymptomatic elevation of ALT levels at week 8, but their ALT levels subsequently returned to the normal range at weeks 10 to 12.^14^ In our study, 12 of the recruited participants had comorbid chronic kidney failure, and 8 of them were required to undergo hemodialysis regularly. There was no significant statistical difference between cirrhosis and noncirrhosis during the course of treatment or follow‐up period (Figure 2). This finding was comparable with recent publication in Japan,^15^ and an SVR12 rate of 100% was established in this subgroup.

This study analyzed the clinical efficacy and safety of EBR/GZR after its launch in Taiwan. The research framework and efficacy analysis results were suitable for comparison with the TRIO research. The results of the present study indicate that EBR/GZR possesses excellent clinical efficacy; the overall SVR12 rate was 98%, and the SVR12 rate of those in the cirrhosis group was 98.7%. Finally, the results demonstrated that EBR/GZR also exhibited excellent efficacy in patients who had received PEG‐IFN plus RBV treatment but failed to respond to it (SVR12, 96.3%). Our study had the following limitations. First, this was a retrospective study according to ICD‐10‐CM codes and medical research database, thus minor adverse events might have been missed and inappropriate decision for DAAs treatment because that recorded and treated by individual physicians. Second, the inappropriate record of the adverse events might result in an inadequate analysis of the result of drug‐drug interaction and safety. Third, the number of patients was limited especially in GT 1a. Thus, a large‐scale study is necessary in real‐world in the future.

## 5 CONCLUSIONS

This study confirmed that EBR/GZR is safe and effective for treating patients with HCV GT 1. An overall SVR rate as high as 98% was achieved. The medication‐related side effects reported in most patients were mild. For patients with ALT levels >10 × ULN after the administration of EBR/GZR, the physicians stopped the EBR/GZR, as recommended. The therapeutic effects are unrelated to factors such as sex, HCV RNA viral load before treatment, and medical history of liver cirrhosis.

## CONFLICT OF INTERESTS

The authors declare that there are no conflict of interests.

## AUTHOR CONTRIBUTIONS

CWH designed and supervised the study. TCT collected clinical data and interpreted the data. TCT and STD performed statistical analysis. TCT and CWH drafted the manuscript. TCT, STD, and CWH approved the final manuscript.
